# Comparison of factors influencing the willingness to donate biospecimens among guardians of children with cancer and adult cancer patients

**DOI:** 10.1002/cam4.4544

**Published:** 2022-02-03

**Authors:** Hongxiang Gao, Baige Cao, Nan Dang, Song Gu, Min Xu, Bin Ji, Yiqi Shi, Shijian Liu, Congrong Wang

**Affiliations:** ^1^ Department of Pediatric General Surgery Shanghai Children’s Medical Center School of Medicine Shanghai Jiao Tong University Shanghai China; ^2^ Department of Endocrinology & Metabolism Shanghai Fourth People's Hospital School of Medicine Tongji University Shanghai China; ^3^ Department of Oncology Shanghai Ninth People’s Hospital Shanghai Jiao Tong University School of Medicine Shanghai China; ^4^ Department of Operating Room Shanghai Children’s Medical Center School of Medicine Shanghai Jiao Tong University Shanghai China; ^5^ Clinical Research Center for Cell Therapy Shanghai East Hospital Tongji University Shanghai China; ^6^ Department of Clinical Epidemiology and Biostatistics Pediatric Health Advocacy Institute Shanghai Children’s Medical Center School of Medicine Shanghai Jiao Tong University Shanghai China; ^7^ School of Public Health Shanghai Jiao Tong University School of Medicine Shanghai China

**Keywords:** adult cancer patient, biospecimen donation, cancer research, childhood cancer patient

## Abstract

**Background:**

This study examined and compared the attitudes and willingness of guardians of children with cancer and adult cancer patients toward donating biospecimens and clinical data for cancer research.

**Methods:**

We conducted a cross‐sectional study among guardians of children with cancer (Guardian group) from Shanghai Children's Medical Center and adult cancer patients (Adult group) from Shanghai Ninth People's Hospital between February 1, 2019, and January 31, 2020. Participants’ demographic data, willingness, and motivations for biospecimen donation were collected and analyzed.

**Results:**

Of 670 participants, 90.8% (318/350) in the Guardian group and 88.1% (282/320) in the Adult group completed the questionnaire. Most participants were willing to donate residual tissue samples (92.8% in the Guardian group vs. 79.4% in the Adult group, *p* ^ψ^ = 0.032) and clinical data (94.0% vs. 72.3%, *p* ^ψ^ < 0.001) for medical research. Logistic regression analysis indicated that only child status (odds ratio [OR] = 0.140, *p* = 0.02), history of blood donation (OR = 4.467, *p* = 0.019) in the Guardian group, education (OR = 0.387, *p* = 0.037), and history of blood donation (OR = 2.556, *p* = 0.016) in the Adult group were significantly associated with participants’ willingness to donate biospecimens. The primary motivation for donation was helping other patients with cancer (65.4% vs. 24.5%, *p* ^ψ^ < 0.001). The major barriers to donation were the potential to cause physical discomfort (61.0% vs. 64.9%, *p* ^ψ^ = 0.032).

**Conclusions:**

Guardians of children with cancer were more willing to donate biospecimens than adult cancer patients in China. It is essential to promote awareness of biospecimens donation, especially in adult cancer patients.

## Graphical abstract

The only child and history of blood donation in the Guardian group, and the education level and history of blood donation in the Adult group were significantly correlated with the participants’ willingness to donate biospecimens. The most common motivation for donation was helping other patients with cancer. The main barriers to donation were potential to cause physical discomfort.

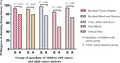

**Lay summary**

This is a cross‐sectional study examining and comparing the attitudes and willingness among guardians of children with cancer and adult cancer patients toward donating biospecimens for cancer research between February 1, 2019, and January 31, 2020. We found that 92.8% of participants in the Guardian group and 79.4% of the participants in the Adult group were willing to donate residual tissue samples. Only child status, history of blood donation in the Guardian group, and education and history of blood donation in the Adult group were significantly associated with participants’ willingness. The primary motivation for donation was helping other patients with cancer, and the major barrier to donation was the potential to cause physical discomfort.

## 1 INTRODUCTION

Human biological specimens, such as blood, tissues, urine, and other body fluids, as well as related clinical data, are important resources for research of cancers and their genetic and physiological causes.^1^, ^2^ Genetic analysis of cancer has become more widespread as the collection of biological specimens has become more common in cancer research. However, some ethical and legal issues still exist to access children's biospecimens for cancer research.^3^

In general, informed consent is provided by the guardian or legal guardian on behalf of the child for children engaged in research in China. The results of genetic research may cause great psychological pressure on guardians if disease‐related gene mutations are identified in their children's biospecimens. Furthermore, if studies reveal children who are genetically inclined to develop certain diseases, it could harm children's access to insurance and education, resulting in discrimination due to their health situation and their families.^4^, ^5^

Recently, a few studies have assessed the attitudes of adult patients and the general population toward cancer research using donated samples, and suggested that factors including age, gender, education level, and history of hospitalization are associated with patients’ willingness to donate biospecimens for research.^4^, ^6^ However, there are limited studies comparing adult cancer patients and children's guardian’ willingness to donate biospecimens for cancer research.

This study aimed to examine and compare the attitudes and willingness of guardians of children with cancer and adult cancer patients toward donating biospecimens and clinical data for cancer research. Understanding participants’ attitudes and motivations for biospecimens donation will help us to improve strategies for obtaining biospecimens and promote the development of medicine.

## 2 METHODS

### 2.1 Study design

**FIGURE 1:**
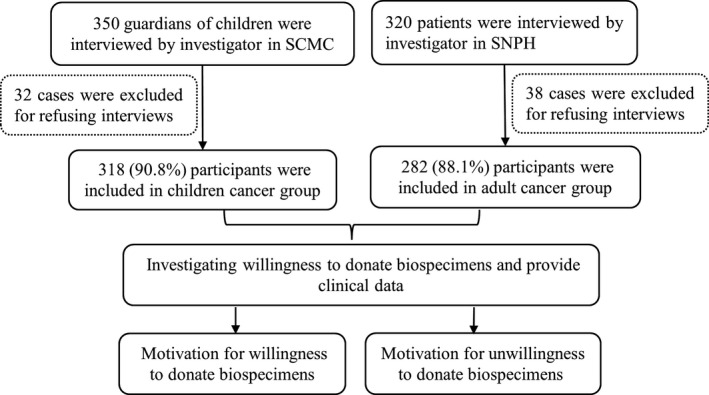
A cross‐sectional survey of biospecimens donation. SCMC, Shanghai Children's Medical Center; SNPH, Shanghai Ninth People's Hospital

We conducted a cross‐sectional survey of the attitudes and perceptions of guardians of children with cancer (Guardian group) and adult cancer patients (Adult group) toward the donation of biological specimens for medical research. Participants in the Guardian group were enrolled from the general surgery inpatient department at the Shanghai Children's Medical Center (SCMC). Participants in the Adult group were enrolled from the Department of Oncology at Shanghai Ninth People's Hospital (SNPH), from February 1, 2019, to January 31, 2020. The investigation flowchart is shown in Figure 1. The inclusion criteria for participation in the Guardian group were as follows: (1) had a legal guardianship relationship with the child diagnosed with cancer; (2) able to read and communicate in Mandarin; and (3) being aged 20 (female) or 22 (male) or above, because China's marriage law sets the legal marriage age at 22 for men and 20 for women.^7^ The inclusion criteria for participation in the Adult group were as follows: (1) diagnosed with cancer; (2) able to read and communicate in Mandarin; and (3) ≥18 years old.

### 2.2 Interview guide

The interviewers (three in SCMC and three in SNPH) were trained and instructed on the objectives and details of the study and to comply with the interview guide and interview procedures. Eligible participants were invited to a separate interview room in the inpatient department for a face‐to‐face interview, and written informed consent was obtained from all participants prior to the interview. At the beginning of the interview session, the interviewer collected and recorded the sociodemographic information of the participants. The main interview session included the following questions: Are you willing to donate your (your child's) biospecimens for cancer research? If you are willing to donate biospecimens, what types of biospecimens are you willing to donate? What are your motivations for willing to donate biospecimens? Why are you unwilling to donate biospecimens? Do you wish to be informed of the general research results of your biospecimens? Is there anything else you would like to add before we finish up?

### 2.3 Study questionnaire

The design of the questionnaire was based on previous related research,^7^, ^8^ focusing on the similarities and differences between guardians’ and adult patients’ demographic information and attitudes about donating biospecimens for medical research. The questionnaire was tested in a pilot survey of 30 participants in each group to ensure that participants fully understood the content of the survey and made clear choices. The primary language of questionnaire was Chinese, with a matching English version.

The questionnaire comprised three domains: the first domain comprised the demographic information of participants, gender, age, nationality, education level, only child status, religiosity, insurance, place of residence, history of blood donation, and family history of cancer; the second domain was participants’ willingness to participate in biospecimen research, including willingness to donate residual tissue samples, residual blood and marrow, urine, saliva and feces, extra blood, and the willingness to provide clinical data for research purposes; and the third domain focused on participants’ motivations and concerns about donating biospecimens for medical research, including benefit to their future treatment or their family, helping other patients with cancer, contributing to medical developments, feel embarrassed to decline a request for a donation, and other reasons (explained briefly).^7^ To investigate the motivations of unwillingness to donate biospecimens, all participants were asked to select one or more of the following six options: (1) potential to cause physical discomfort, (2) worry about personal privacy being disclosed, (3) absence of financial benefit, (4) do not trust the hospital or the researchers, (5) worry about researchers profiting from the biospecimens, and (6) other reasons (explained briefly).^7^

### 2.4 Data analysis

The participants’ characteristics were presented as frequency counts and percentages. The Mann–Whitney test and ANCOVA were used for group comparison and controlled for age, gender, nationality, education, place of residence, and only child status. Pearson χ^2^ tests were performed to demonstrate the relationship between attitudes toward donating residual tissue and demographic variables in the Guardian group and the Adult group, respectively. Univariate logistic regression was used to examine the associations between willingness to participate in biospecimen research and sociodemographic factors. Variables identified as statistically significant at the level of 0.05 in the univariate analyses were included in the multivariate analysis. Multiple logistic regression was used to examine whether demographic factors were associated with participants’ willingness to donate biospecimens.^7^ Odds ratio (OR) and 95% confidence interval (CI) were reported. All tests were two‐tailed, and statistical significance was set at *p* < 0.05. All analyses were performed using SPSS (version 22.0; SPSS Inc., Chicago, Illinois, USA).

## 3 RESULTS

### 3.1 Demographic characteristics

A total of 670 participants were invited to complete the anonymous questionnaire, with a response rate of 90.8% (318/350) in the Guardian group and 88.1% (282/320) in the Adult group. The interviewees’ demographic characteristics are summarized in Table 1. Men accounted for 56.9% of the study population (31.6% in the Guardian group vs. 58.2% in the Adult group, *p* < 0.001). The average age of the subjects was 47.6 ± 17.0 years in total; the age of the guardians was significantly lower than that of the adults (35.0 ± 6.7 years in the Guardian group vs. 61.7 ± 13.5 years in the Adult group, *p* < 0.001). Approximately 97.0% of the participants were Han Chinese (95.3% in the Guardian group vs. 98.9% in the Adult group, *p* = 0.009). More guardians than adults achieved a college or graduate degree (51.2% vs. 18.8%, *p* < 0.001). A higher percentage of guardians was only child than adults (43.1% in the Guardian group vs. 9.2% in the Adult group, *p* < 0.001). Data concerning health insurance, religiosity status, place of residence, and family history of cancer were comparable between the two groups (*p* > 0.05).

**TABLE 1:** Demographic characteristics of participants

Characteristic	Total (*n* = 600), *n* (%)	Guardian group (*n* = 318), *n* (%)	Adult group (*n* = 282), *n* (%)	*p*
Gender				**<0.001**
Male	289 (48.2)	125 (31.6)	164 (58.2)	
Female	311 (51.8)	193 (68.4)	118 (41.8)	
Age (years)				**<0.001**
20–40	198 (33.0)	184 (57.9)	14 (5.0)	
41–60	245 (40.8)	130 (40.8)	115 (40.8)	
>61	157 (26.2)	4 (1.3)	153 (54.2)	
Nationality				**0.009**
Chinese Han	582 (97.0)	303 (95.3)	279 (98.9)	
National minority	18 (3.0)	15 (4.7)	3 (1.1)	
Education				**<0.001**
Below high school	138 (23.0)	68 (21.4)	70 (24.8)	
High school	246 (41.0)	87 (27.4)	159 (56.4)	
College or graduate degree	216 (36.0)	163 (51.2)	53 (18.8)	
Only child status				**<0.001**
Yes	163 (27.2)	137 (43.1)	26 (9.2)	
No	437 (72.8)	181 (56.9)	256 (90.8)	
Religiosity				0.672
Yes	76 (12.7)	42 (13.2)	34 (12.1)	
No	524 (87.3)	276 (86.8)	248 (87.9)	
Insurance				0.483
Yes	557 (92.8)	293 (92.1)	264 (93.6)	
No	43 (7.2)	25 (7.9)	18 (6.4)	
Place of residence				**0.003**
Urban area	366 (61.0)	176 (55.3)	190 (67.4)	
Rural area	234 (39.0)	142 (44.7)	92 (32.6)	
History of blood donation				0.124
Yes	297 (49.5)	148 (46.5)	149 (52.8)	
No	303 (50.5)	170 (53.5)	133 (47.2)	
Family history of cancer				0.376
Yes	47 (7.8)	22 (6.9)	25 (8.9)	
No	553 (92.2)	296 (93.1)	257 (91.1)	

Note

Guardian group: guardians of children with cancer, Adult group: adult cancer patients. Bold shows *p* ≤ 0.05.

### 3.2 Participants’ willingness to donate biospecimens

**FIGURE 2:**
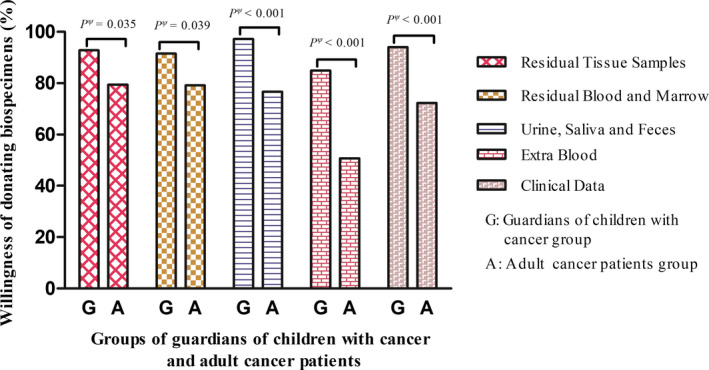
Participants’ willingness toward donating biospecimens. Guardian group: guardians of children with cancer, Adult group: adult cancer patients. ^ψ^ *p* value adjusted for age, gender, nation, education, place of residence, and only child status between the Guardian group and the Adult group by ANCOVA analysis

Participants’ attitudes toward donating biospecimens for research purposes are summarized in Figure 2, participants in the Guardian group were more willing to donate biospecimens than those in the Adult group. Among the 600 participants, the percentage of willingness to donate residual tissue samples; residual blood and marrow, urine, saliva and feces, extra blood, and providing clinical data was 86.5%, 85.7%, 87.5%, 68.8%, and 83.8%, respectively. All of the percentages of the guardians of children with cancer were significantly higher than those of adult patients (*p* < 0.05) (Figure 2).

### 3.3 Factors associated with willingness to donate residual tissue samples

The guardians’ willingness to donate residual tissue was associated with age (*p* < 0.001), only child status (*p* < 0.001), insurance (*p* = 0.01), and history of blood donation (*p* = 0.004), while adult cancer patients’ willingness to donate residual tissue was related to age (*p* = 0.033), education (*p* = 0.007), religiosity (*p* = 0.024), and history of blood donation (*p* < 0.001), both groups were associated with age and history of blood donation (Table 2).

**TABLE 2:** Participants’ willingness to donate residual tissue samples

Characteristic	Guardian group				Adult group			
	Agree (*n* = 295), *n* (%)	Disagree (*n* = 23), n (%)	*p*	OR (95% CI)	Agree (*n* = 224), *n* (%)	Disagree (*n* = 58), *n* (%)	*p*	OR (95% CI)
Gender			0.385				0.202	
Male	114 (91.2)	11 (8.8)		0.479 (0.177–1.292)	126 (76.8)	38 (23.2)		0.545 (0.264–1.121)
Female	181 (93.8)	12 (6.2)		1.000	98 (83.1)	20 (16.9)		1.000
Age (years)			**<0.001**				**0.033**	
20–40	167 (90.8)	17 (9.2)		0.910 (0.787–1.053)	13 (92.9)	1 (7.9)		1.042 (0.990–1.097)
41–60	128 (98.5)	2 (1.5)		1.060 (0.662–1.082)	98 (85.2)	17 (14.8)		0.978 (0.932–1.027)
>61	0 (0.0)	4 (100.0)		1.000	113 (74.5)	40 (25.5)		1.000
Nationality			0.268				0.376	
Chinese Han	280 (92.4)	23 (7.6)		3.616 (0.597–11.885)	221 (79.2)	58 (20.8)		0.945 (0.049–8.204)
National minority	15 (100.0)	0 (0.0)		1.000	3 (100.0)	0 (0.0)		1.000
Education			0.118				**0.007**	
Below high school	66 (97.0)	2 (3.0)		0.679 (0.347–1.328)	60 (85.7)	10 (14.3)		0.387 (0.158–0.946)
High school	77 (88.5)	10 (11.5)		0.733 (0.175–3.065)	116 (73.0)	43 (27.0)		1.395 (0.689–2.823)
College or graduate degree	152 (93.2)	11 (6.8)		1.000	48 (90.6)	5 (9.4)		1.000
Only child status			**<0.001**				0.232	
Yes	119 (86.9)	18 (13.1)		0.140 (0.040–0.492)	23 (88.5)	3 (11.5)		1.133 (0.248–5.188)
No	176 (97.2)	5 (2.8)		1.000	201 (78.5)	55 (21.5)		1.000
Religiosity			0.981				**0.024**	
Yes	39 (92.9)	3 (7.1)		1.337 (0.347–5.155)	32 (94.1)	2 (5.9)		0.348 (0.068–1.779)
No	256 (92.8)	20 (7.2)		1.000	192 (77.4)	56 (22.6)		1.000
Insurance			**0.010**				0.858	
Yes	275 (93.9)	18 (6.1)		3.131 (0.866–11.332)	210 (79.5)	54 (20.5)		2.862 (0.572–14.316)
No	20 (80.0)	5 (20.0)		1.000	14 (77.8)	4 (22.2)		1.000
Place of Residence			0.323				0.063	
Urban area	161 (91.5)	15 (8.5)		0.812 (0.297–2.218)	145 (76.3)	45 (23.7)		0.807 (0.340–1.917)
Rural area	134 (94.4)	8 (5.6)		1.000	79 (85.9)	13 (14.1)		1.000
History of blood donation			**0.004**				**<0.001**	
Yes	144 (97.3)	4 (2.7)		4.467 (1.281–15.579)	146 (98.0)	3 (2.0)		2.556 (1.194–5.471)
No	151 (88.8)	19 (11.2)		1.000	78 (58.6)	55 (41.4)		1.000
Family history of cancer			0.727				0.267	
Yes	20 (90.9)	2 (9.1)		0.360 (0.065–1.976)	22 (88.0)	3 (12.0)		1.259 (0.253–6.265)
No	275 (92.9)	21 (7.1)		1.000	202 (78.6)	55 (21.4)		1.000

Note

Guardian group: guardians of children with cancer, Adult group: adult cancer patients. Bold shows *p* ≤ 0.05.

Logistic regression analysis indicated that only child status (OR = 0.140, 95% confidence interval [CI]: 0.040–0.492, *p* = 0.02) and history of blood donation (OR = 4.467, 95% CI: 1.281–15.579, *p* = 0.019) in the Guardian group, and education (below high school: OR = 0.387, 95% CI: 0.158–0.946, *p* = 0.037; high school: OR = 1.395, 95% CI: 0.689–2.823, *p* = 0.355) and history of blood donation (OR = 2.556, 95% CI: 1.194–5.471, *p* = 0.016) in the Adult group were significantly associated with participants’ willingness to donate residual tissue samples, respectively (Table 2).

### 3.4 Motivations for donating biospecimens

Participants’ motivations for willingness to donate biospecimens for research purposes are summarized in Table 3; the percentage of participants who benefited from future treatment, their family, help other patients with cancer, and contribute to medical developments was 35.8%, 17.2%, 46.2%, and 25.5%, respectively. The percentages of willingness to donate biospecimens in the Guardian group were significantly higher than that in the Adult group (*p* < 0.001 in the unadjusted model, Table 3).

**TABLE 3:** Motivation for donating biospecimens

Characteristic	Total (*n* = 600), *n* (%)	Guardian group (*n* = 318), *n* (%)	Adult group (*n* = 282), *n* (%)	*p*	*p* ^a^
Willing to donate biospecimens					
Benefit to their future treatment				**<0.001**	**0.023**
Yes	215 (35.8)	150 (47.2)	65 (23.0)		
No	385 (64.2)	168 (52.8)	217 (77.0)		
Benefit to their family				**<0.001**	**<0.001**
Yes	103 (17.2)	77 (24.2)	26 (9.2)		
No	497 (82.8)	24 (72.8)	256 (90.8)		
Helping other patients with cancer				**<0.001**	**<0.001**
Yes	277 (46.2)	208 (65.4)	69 (24.5)		
No	323 (53.8)	110 (34.6)	213 (75.5)		
Contributing to medical developments				**<0.001**	0.173
Yes	153 (25.5)	106 (33.3)	47 (16.7)		
No	447 (74.5)	212 (66.7)	235 (83.3)		
Unwilling to donate biospecimens					
Potential to cause physical discomfort				0.325	**0.032**
Yes	377 (62.8)	194 (61.0)	183 (64.9)		
No	223 (37.2)	124 (39.0)	99 (35.1)		
Worry about personal privacy being disclosed				**< 0.001**	0.210
Yes	184 (30.7)	122 (38.4)	62 (22.0)		
No	416 (69.3)	196 (61.6)	220 (78.0)		
Absence of financial benefit				**<0.001**	**0.007**
Yes	101 (16.8)	33 (10.4)	68 (24.1)		
No	499 (83.2)	285 (89.6)	214 (75.9)		
Do not trust in the hospital or the researchers				**0.001**	**0.011**
Yes	43 (7.2)	33 (10.4)	10 (3.5)		
No	557 (92.8)	285 (89.6)	272 (96.5)		
Worry about researchers profiting from the biospecimens				**0.039**	**0.023**
Yes	119 (19.8)	53 (16.7)	66 (23.4)		
No	481 (80.2)	265 (83.3)	216 (76.6)		

Note

Guardian group: guardians of children with cancer, Adult group: Adult cancer patients. Bold shows *p* ≤ 0.05.

^a^ *p* value adjusted for age, gender, nation, education, place of residence, and only child status between the Guardian group and the Adult group by ANCOVA analysis.

Participants’ motivations for unwillingness to donate biospecimens for research purposes are summarized in Table 3. More than half (62.8%) of the participants thought it may cause physical discomfort in total and 61.0% in the Guardian group versus 64.9% in the Adult group, *p* ^ψ^ = 0.032. Approximately 30.7% of participants worried about personal privacy being disclosed in total and 38.4% in the Guardian group versus 22.0% in the Adult group, *p* ^ψ^ = 0.210. About 16.8% of participants thought it did not have self‐benefit; 10.4% in the Guardian group versus 24.1% in the Adult group, *p* ^ψ^ = 0.007. Only 7.2% of participants did not trust in the hospital or the researchers; 10.4% in the Guardian group versus 3.5% in the Adult group, *p* ^ψ^ = 0.011. Lastly, 19.8% of participants worried about researchers profiting from the biospecimens; 16.7% in the Guardian group versus 23.4% in the Adult group, *p* ^ψ^ = 0.006. None of the participants reported any other reasons.

### 3.5 Participants’ attitudes toward research results of biospecimens

Among the 600 participants, 88.3% expressed a desire to obtain the research results of biospecimens (i.e., general research results), 94.0% in the Guardian group versus 81.9% in the Adult group, *p* < 0.001, *p* ^ψ^ = 0.024 after adjustment.

## 4 DISCUSSION

The overall results of the current study revealed that 86.5% of the participants were willing to donate residual tissue samples for cancer research, a percentage close to those previously reported for patients with cancer (87.1% for residual tissue and 83.3% for residual blood after diagnosis).^9^ More than 90% of participants in the Guardian group were willing to donate biospecimens except extra blood, which was higher than the Adult group, and a previous study (88.6% of guardians agreed to donate their children's biospecimens to a biobank).^10^ Of the four different types of biospecimens, participants were less likely to donate extra blood for medical research in both groups.

Previous studies had confirmed that gender, age, place of residence, education, and other demographic factors, were associated with participants’ willingness to donate biospecimens for research.^11^, ^12^, ^13^ Our findings showed that participants’ willingness to donate biospecimens was significantly associated with only child status, education level, and history of blood donation. Specifically, participants with only child status in the Guardian group and below high school degree in the Adult group were associated with negative attitudes toward residual tissue samples donation. Participants with a history of blood donation in both groups were more likely to donate residual tissue samples for medical research.

The characteristics of gender, age, education, and only child status differed between adults and guardians in this study. In China, mothers have more guardianship responsibilities than fathers in a family, so the percentage of female guardians who participated in this study (68.4%) was higher than that of the adult patients (41.8%). Because the incidence of cancer in adults was relatively higher in older people,^14^ 95% of participants in the Adult group were over 40 years old in this study. Thus, the adult cancer patients were older than the guardians of children with cancer. In addition, the guardians of children with cancer have higher education levels than the adult cancer patients.

Due to the one‐child family planning policy promulgated in 1979 in China, there has been an increasing number of families with only one child in the past 40 years, so the percentage of only child status is higher in the Guardian group than in the Adult group. In China, only‐child families have a closer parent‐child relationship than non‐only‐child families, which means guardians of only‐child families dedicate more parent‐child time, energy, and attention to them.^15^ It is reasonable that guardians with only one child were more reluctant to donate their children's tissue samples for cancer research. Qiu et al. reported in 2018 that guardians with only one child were less likely to donate biospecimens for medical research,^16^ which was consistent with our findings.

Our findings indicated that adult cancer patients with lower education level were less likely agree to donate biospecimens, these patients were less clear on “biospecimens donation,” which was consistent to those studies that involve the general population and patients in China and other countries.^17^ Ahram et al. reported that willingness to participate in biobanks was associated with higher levels of education, higher incomes, and younger age.^18^

A history of voluntary or therapeutic blood donation may stimulate participants’ passion to donate biospecimens for cancer research in both groups. Participants with a history of blood donation were more aware of the importance of biological samples for cancer research and were more likely to be directly involved in medical research.^19^

In our study, participants in the Guardian group were more positively motivated to donate biospecimens than those in the Adult group. Most study participants said that they would probably consider donating biospecimens if there was a direct benefit to their health or that of their family.^20^, ^21^ As it turns out, this is an important reason for participants who were in favor of scientific research and the donation of biospecimens. Furthermore, participants’ primary motivation for donating biospecimens was a potential benefit for other cancer patients. Participants were more likely to understand and empathize with other cancer patients after experiencing difficulties in diagnosis, surgery, and postoperative treatment; therefore, they were more willing to donate biospecimens for cancer research and medical development.^22^

Previous studies have reported that common barriers to biospecimens donation include the potential to cause physical discomfort, risk of privacy disclosure, cultural beliefs about biospecimens, absence of financial benefits, and distrust of researchers.^12^, ^23^, ^24^ The primary barrier for participants’ reluctance to donate biospecimens was the physical discomfort that the donation process could cause to patients in both groups in this study. The secondary barrier for participants was worrying about personal privacy being disclosure. In this study, 36.8% of the only child participants were worried about privacy disclosure, which was higher than that of the non‐only child participants (28.4%), *p* = 0.046. Cancer patients are increasingly concerned about the privacy and confidentiality of their disease status, as well as the possible consequences of donating their biospecimens to medical research.^25^ Building public trust and confidence is an important step to ensure that biospecimens are available for long‐term medical research.

A majority of participants expressed the expectation to receive the research results of biospecimens, especially the guardians of children with cancer. Many participants believed that knowing genetic information about themselves or their family members would prompt people to be more concerned about preventive measures, adopt a healthier lifestyle, or improve the chances of getting better treatment for cancer.^26^, ^27^ Furthermore, obtaining research results has been an influential factor in participants’ decision to donate biospecimens, and researchers are obligated to provide genetic results to donors and/or their families.^28^, ^29^

## 5 STRENGTHS AND LIMITATIONS

The advantages of this study were high‐quality face‐to‐face interviews with high response rates, and the privacy of participants was protected during the survey process. No previous study has focused on the similarities and differences between guardians’ and adult cancer patients’ attitudes and willingness to donate biospecimens and clinical data for cancer research. However, this study has a few limitations. First, there are differences in tumor types and severity between children and adults; guardians of children with cancer in the surgery inpatient department may have a more intuitive understanding of the biospecimens than patients in the oncology inpatient department, which may cause potential bias. Second, we only surveyed the participants’ willingness to donate biospecimens, which may or may not translate into actual behavior. Third, participants were emotional, shocked, and confused about the cancer diagnosis, which might have a positive impact on their willingness to donate biospecimens for cancer research. However, in our study, participants were not newly diagnosed but were in a stable medical condition. Thus, emotion might have a negligible influence on participants’ willingness to donate biospecimens.

## 6 CONCLUSION

This study provides new empirical data on the attitudes toward biospecimens donation among guardians of children with cancer and adult cancer patients. Guardians of children with cancer were more willing to donate biospecimens for cancer research than adult cancer patients in China. It is necessary to establish awareness campaigns to enhance the public's understanding of biospecimens donation, especially in adult cancer patients.

## ETHICS STATEMENT

This study was approved by the institutional review boards of Shanghai Children's Medical Center and the institutional review boards of Shanghai Ninth People's Hospital.

## INFORMED CONSENT STATEMENT

All participants gave written informed consent prior to engage in this study.

## CONFLICT OF INTEREST

None.

## AUTHOR CONTRIBUTION

Hongxiang Gao: Conceptualization, investigation, writing—original draft, and writing—review and editing. Baige Cao: Formal analysis and writing—review and editing. Nan Dang: Data curation, investigation, formal analysis, and resources. Song Gu: Data curation and resources. Min Xu: Conceptualization and resources. Bin Ji: Data curation and investigation. Yiqi Shi: Data curation and investigation. Shijian Liu: Conceptualization, formal analysis, project administration, supervision, and writing—review and editing. Congrong Wang: Conceptualization, funding acquisition, and writing—review and editing.

## Data Availability

The data that support the findings of this study are available from the corresponding author upon reasonable request.
